# Synchronous and Metachronous Breast Malignancies: A Cross-Sectional Retrospective Study and Review of the Literature

**DOI:** 10.1155/2014/250727

**Published:** 2014-04-27

**Authors:** Ambrogio P. Londero, Sergio Bernardi, Serena Bertozzi, Vito Angione, Giuliana Gentile, Cinzia Dri, Antonio Minucci, Filippo Caponnetto, Roberto Petri

**Affiliations:** ^1^Clinic of Obstetrics and Gynecology, University of Udine, 33100 Udine, Italy; ^2^Department of Surgery, AOU “SM della Misericordia,” 33100 Udine, Italy; ^3^Department of Pathology, AOU “SM della Misericordia,” 33100 Udine, Italy

## Abstract

*Objective*. There is increasing interest in patients with metachronous (MBC) and synchronous breast cancer (SBC). The objective of this study was to evaluate the occurrence and outcome of MBCs and SBCs. *Methods*. A retrospective study on women operated in our department for breast cancer between 2002 and 2005 was carried out. Patients were divided into three groups: women with MBC, SBC, and unilateral breast cancer (UBC). Moreover, we performed a meta-analysis of the English literature about multiple breast cancers between 2000 and 2011 taking into consideration their prevalence and overall survival (OS). *Results*. We identified 584 breast cancer patients: 16 women (3%) presented SBC and 40 MBC (7%, second cancer after 72-month follow-up IQR 40–145). Although the meta-analysis showed significant OS differences between MBC or SBC and UBC, we did not observe any significant OS difference among the three groups of our population. Anyway, we found a significant worse disease-free survival in MBC than UBC and a significant higher prevalence of radical surgery in MBC and SBC than UBC. *Conclusions.* Despite the low prevalence of MBC and SBC, the presence of a long time risk of MBC confirms the crucial role of ipsi- and contralateral mammographies in the postoperative follow-up.

## 1. Introduction

The increasing incidence rate of breast cancer (BC) and its long term survival, due to both an improved prognosis and a growing life expectancy, have brought interest in patients with a second primary metachronous (MBC) or synchronous breast cancer (SBC) [1–3]. In particular, BC represents about the 30–50% of all second primary malignancies in women affected by primary BC, who have a two-to-sixfold increased risk of developing a new primary cancer in the contralateral breast during their life [4–6], corresponding to a risk of 0.3–1.0% per year [7–12].

There is uncertainty in the literature whether developing a MBC or SBC influences the outcome. In fact, some studies suggest poor survival while others report similar survival compared to patients with unilateral breast cancer (UBC). In particular, women affected by SBCs seem to have a lower long term survival [13–15], even if this difference is not always significant. Moreover, there is conflicting evidence about the impact of SBCs and MBCs on the management of patients with regard to surgical treatment options, such as the role of prophylactic mastectomy. In fact, these patients undergo more often bilateral mastectomies rather than breast conserving interventions, although there are reports confirming the efficacy of less invasive management in bilateral breast cancer as for unilateral tumors [14, 16].

The objective of this study is to evaluate the occurrence of MBCs or SBCs and their outcome.

## 2. Materials and Methods

### 2.1. Retrospective Study

A retrospective cross-sectional study on women operated in our department for BC between January 2001 and December 2004 was carried out, with a follow-up of at least 5 years, within May 2011. In addition, we selected only women with the first diagnosis of cancer occurring between January 2001 and December 2004. Patients were divided into three groups based on the presence of multiple breast cancers and the time interval between their detection. In particular, according to most of the authors, we considered synchronous all second BCs detected within 6 months from the first diagnosis and located in the contralateral breast [17–19] and metachronous those diagnosed after 6 months from the first BC diagnosis in the contralateral breast or in the same breast but with different histology. The remaining women with a diagnosis of UBCs were considered as a control cohort.

Tumor pathological characteristics include size, histological type, nuclear/histologic grading, multicentricity, eventual presence of peritumoral vascular invasion (PVI), extensive* in situ* component (EIC), axillary lymph node status, lymph nodes extracapsular invasion (ExCp), estrogen (ER) and progesterone receptor (PR) status, HER-2/Neu expression, Mib-1(Ki-67) expression. The surgical specimens were examined fresh and the maximum tumor diameter was recorded after these were fixed in 10% buffered formalin. Histologically, the tumors were classified according to the World Health Organization criteria [20], as modified by Rosen and Oberman [21]. Tumor grade was evaluated following the recommendations of Elston and Ellis [22]. The occurrence of PVI was assessed according to Rosen and Oberman [21]. The expression of ER, PR, Her-2/Neu, and the tumor proliferative fraction (Mib1/Ki67) was evaluated immunohistochemically. ER and PR status and cell proliferation were recorded as percentage value. HER-2/Neu was considered positive when Her-2/Neu test resulted 3+ or 2+ with fish amplification and negative if value was 1+. ExCp was defined as the extracapsular growth of tumor cells, invasion of perinodal fat, or extranodal location of tumor cells. Tumor stage was defined according to the TNM classification of 2009 VII ed. (AJCC/UICC).

Data about patients' familial history were also collected, and, for the purpose of our retrospective study, a positive familial history was defined by the presence of a first- or second-degree relative with breast cancer. Moreover, data was collected about the patients' age, at both the first and the second diagnosis, weight, breast size and density, tobacco smoking habits, the tumor localization (breast side and quadrant), the diagnostic tool (objective examination, mammography, or ultrasonography), and the specific finding at the first and the second diagnosis. Then, we retrieved information also about the treatment received for the first and the second tumor and type of surgery performed.

The three groups (MBC, SBC, and UBC) were compared, taking into consideration the following outcomes: overall survival (OS), disease-free survival (DFS), overall mortality and cancer-related mortality, and locoregional and distant disease recurrence. The study was approved by the local ethics committee.

### 2.2. Meta-Analysis

To perform our meta-analysis we searched in the following sources: Medline, Cochrane database, EBESCO, and Google Scholar. We included in our search only the English literature published between 2000 and 2011 and used the following search keywords: synchronous and metachronous breast cancer, bilateral breast cancer. We took into consideration in our analysis every retrospective or prospective study that evaluated survival in patients affected by MBC, SBC, and UBC. All included studies were observational and we took into consideration only those studies which utilized a Cox proportional hazards regression model for calculating the survival difference among MBC, SBC, and UBC. In addition, we included only articles of which the full text was available for data retrieval. Then, three reviewers independently extracted data from the included studies onto a standard form. The data abstracted were relevant to predetermined measures (prevalence of disease and hazards ratios with 95% confidence interval). The hazard ratio in our meta-analysis was calculated from data obtained from published reports, using methods previously described [23]. Geographic locations, sites of treatment, and time frame for breast cancer diagnosis were recorded, in order to avoid any possible population overlap, and when these features suggested a population overlap between two reports the study with longer follow-up or a larger data set was utilized, while the other was excluded from the pooled analysis. To perform this meta-analysis we took into consideration the MOOSE guidelines [24].

### 2.3. Statistical Analysis

Data was analysed by R (version 2.13.1), considering significant *P* < 0.05. All continuous variables were tested for distribution normality with Kolmogorov-Smirnov test. Monovariate analysis was performed by analysis of variance, Kruskall-Wallis test,* t*-test, or Wilcoxon test in case of continuous variables and chi-square test or Fisher exact test in case of categorical variables. The OS expressed in months was calculated from both the first malignancies and considered for MBCs from both the first and the second diagnosis, taking into consideration the one from the second diagnosis for OS and DFS analysis. The 5-year survival rates were also computed. Differences between the survival Kaplan-Meyer curves for MBC, SBC, and UBC were tested by means of the log-rank test. Moreover, the Cox proportional hazards model was used in the multivariate survival analysis, and the Cohen kappa value (1 indicates a perfect agreement, 0 denotes the lack of agreement, and the* P* value indicates whether the estimated kappa value differs from 0) was used to test the agreement of the investigated tumor characteristics within the same patient. We investigated the agreement also calculating the percentage of tumors within the same subject presenting the same characteristics. In the meta-analysis, a summary statistic was calculated considering, where appropriate, the prevalence or hazards ratio for survival analysis. The random effect model was applied to calculate the pooled estimate, as heterogeneity between studies was expected, a priori and confirmed by Q statistic and I2 index. Furthermore, we used rank correlation test of funnel plot asymmetry to test the presence of any publication bias.

## 3. Results

### 3.1. Population Description and Prevalence of MBC and SBC

Among 736 patients operated for a breast pathology in our department during the study period, we identified 584 breast cancer patients with the first BC diagnosis made during the same period: 16 women (3%) presented SBC and 40 presented MBC (7%). The median time interval between the first and the second primary cancer diagnosis in case of MBC resulted 72 months (IQR 40–120), being the 59% of metachronous cancers diagnosed after the 5th year of follow-up and the 40% after the 10th. We had also one case of SBC which was treated with conservative methods and subsequently developed a MBC with different histology.

Table 1 shows the characteristics of our population. SBCs appeared in a significantly elder population, as well as the second cancer in the MBC group. SBCs had a higher prevalence of familial history of breast cancer than other patients. Moreover, second synchronous and metachronous tumors were more frequently detected through the mammographic follow-up (*P* < 0.05). There was also a significant higher prevalence of hormonal therapies in SBCs than UBCs (*P* < 0.05).

### 3.2. Tumor Characteristics and Outcome

Table 2 describes the tumors characteristics and highlights a higher prevalence of lobular invasive histology among SBCs and MBCs than UBCs, but this is statistically significant only between SBCs and UBCs. No significant difference was observed among the considered groups about the TNM classification at diagnosis.

In Table 3 we see that the first and the second MBCs presented a higher prevalence of grading G3 than UBCs, but this difference was significant only between the first MBCs and UBCs (*P* < 0.05). Furthermore, we observed a nonsignificant lower prevalence of grading G3 in SBCs than UBCs. First MBCs expressed also less frequently estrogen and progesterone receptors than UBCs (*P* < 0.05), and they had a significantly lower prevalence of multifocality (*P* < 0.05). SBCs presented the same prevalence of estrogen and progesterone receptors expression as UBCs but a significantly higher prevalence of multifocality and lymph node extracapsular invasion than UBCs (*P* < 0.05).

The first and the second SBCs presented the same histological type in 44% of cases (7/16) (kappa 0.138, *P*  0.225), grading in 69% (11/16) (kappa 0.223, *P* 0.232), ER positivity in 69% (11/16) (kappa 0.200, *P* 0.261), and PR positivity in 69% (11/16) (kappa 0.200, *P* 0.261). The first and the second MBCs presented the same histological type in 42% of cases (17/40) (kappa 0.069, *P* 0.151), grading in 42% (17/40) (kappa 0.006, *P* 0.483), ER positivity in 40% (16/40) (kappa 0.029, *P* 0.585), and PR positivity in 45% (18/40) (kappa 0.156, *P* 0.097). In addition, the second metachronous tumors were ipsilateral in 13% (5/40) of cases and contralateral in 85% (34/40), and one case happened after previous bilateral synchronous cancers 2% (1/40).

We found also a significantly higher prevalence of mastectomy for the second MBCs than UBCs (52% versus 36%, *P* < 0.05) and for any SBCs than UBCs (56% versus 36%, *P* < 0.05), being the 50% of SBCs treated with bilateral mastectomy.

**Figure 1:**
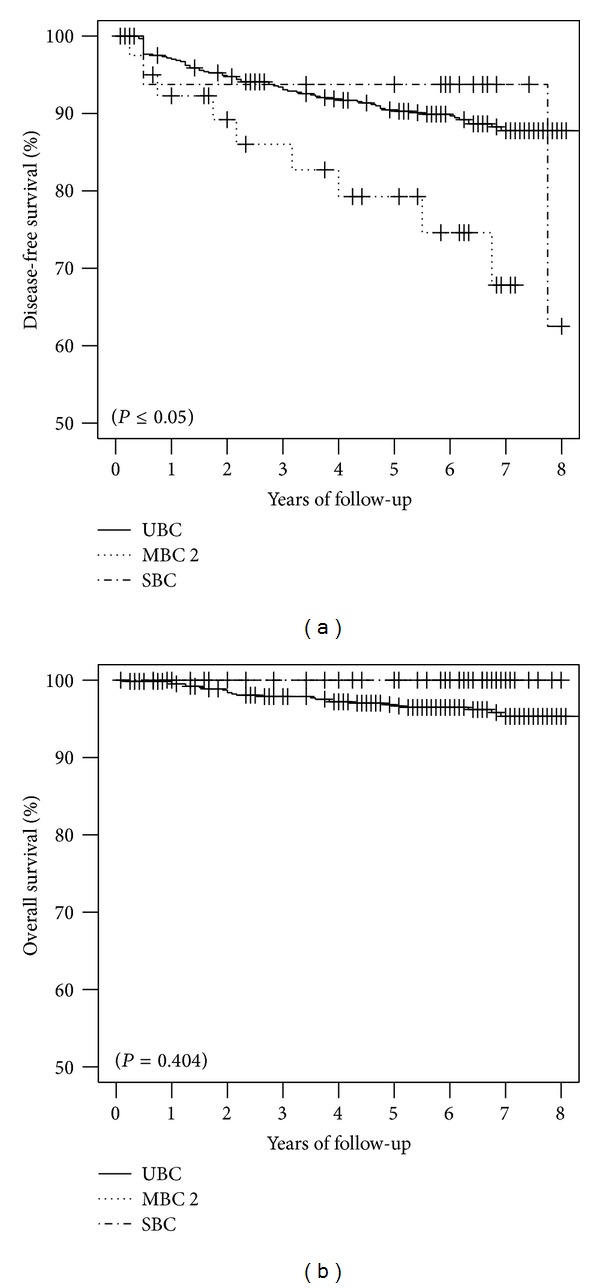
Kaplan-Meyer curves and *P* values calculated by log-rank test. We have on the left side the curves of disease free survival and on the right side the curves of overall survival.

Analyzing the OS, no statistically significant difference was observed between the three groups (Figure 1). Anyway, women affected by SBCs and MBCs had lower prevalences of death for neoplasm than UBCs. Considering the DFS, locoregional relapse or distant metastasis happened more frequently during follow-up of MBCs and SBCs than UBCs. Anyway, in the Kaplan-Meier curves of DFS the log-rank test was statistically significant only between MBCs and UBCs (*P* < 0.05) (Figure 1). This difference was also statistically significant using the monovariate, multivariate, and Cox proportional hazards model with adjustment for tumor dimensions, histology, lymph nodes status, age at diagnosis, and BMI (the HR are reported in Table 4). The 5-year OS in UBCs was 97% (95% CI: 95%–98%), while among MBCs and SBCs it was 100% (95% CI: 100%-100%). In addition, the 5-year DFS was 90% (95% CI: 88%–93%) in UBCs, 79% (95% CI: 66%–94%) in second MBCs, and 94% (95% CI: 83%–100%) in SBCs.

A shorter time interval between the first and the second tumor in women affected by MBCs did not predict a worse OS or DFS but resulted to be associated with a significantly lower hormone receptors positivity (*P* < 0.05) of the second MBC. Moreover, patients affected by MBCs, appearing within 47 months of follow-up, resulted to be older and to have a higher prevalence of lobular invasive cancers than patients with MBCs appearing after 47 months (*P* < 0.05) (Table 5). Furthermore, we observed a higher prevalence of G3 grading, peritumoral vascular invasion, extended in situ component, and multifocality among second MBCs appearing within 47 months of follow-up than UBCs (p n.s.) (Table 5).

### 3.3. Review of the Literature and Meta-Analysis

We found 1840 pertinent abstracts published between January 2000 and August 2011. Then, we selected and retrieved 24 full articles to be candidate for the analysis, 7 of which resulted to be appropriate for the meta-analysis comparing SBCs with UBCs and 6 comparing MBCs with UBCs.  Table 6 summarized the data characteristics of the included studies [7, 15, 17–19, 25, 26]. Flow diagram depicting selection of articles for review is included as Supplemental Figure 1 in the Supplementary Materials available online at http://dx.doi.org/10.1155/2014/250727 and more details about included and excluded studies are reported in Supplemental List 1.

**Figure 2:**
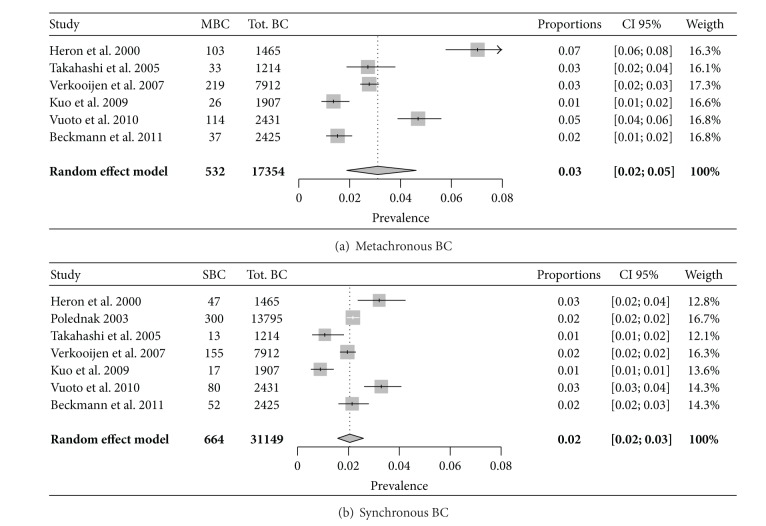
Meta-analysis of SBCs and MBCs prevalence. (a) Heterogeneity: *I*2 = 95.9%, *Q* = 121.1, df = 5, and *P* < 0.0001; (b) heterogeneity: *I*2 = 87.7%, *Q* = 48.7, df = 6, and *P* < 0.0001.

**Figure 3:**
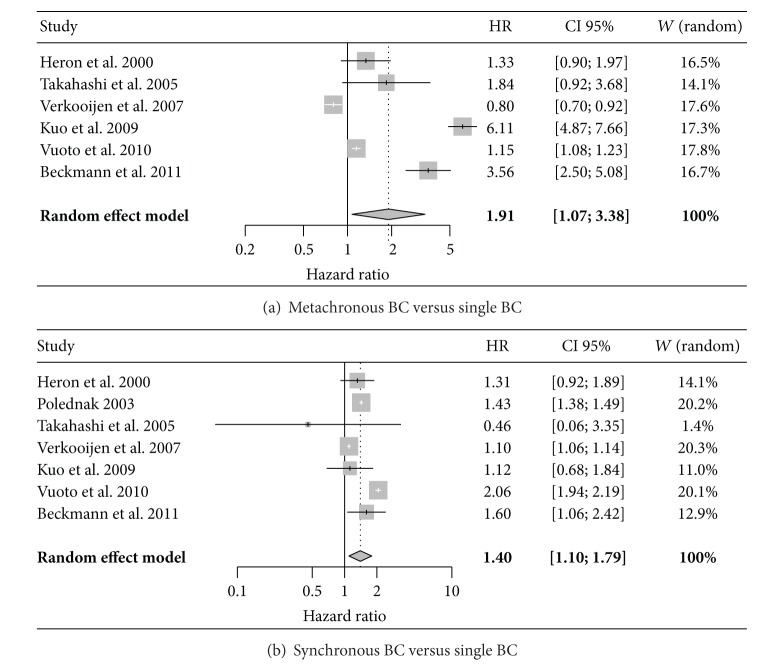
Meta-analysis of SBCs and MBCs overall survival statistics. (a) Heterogeneity: *I*2 = 99%, *Q* = 477.6, df = 5, and *P* < 0.0001; (b) heterogeneity: *I*2 = 98.3%, *Q* = 344.8, df = 6, and *P* < 0.0001.

Through the meta-analysis, the prevalence of MBCs and SBCs was, respectively, 3% (CI 95%: 2–5%) and 2% (2-3%) (Figure 2). Moreover, considering the included studies, MBCs and SBCs presented a significantly unfavorable outcome (in terms of OS) in comparison to UBCs (Figure 3).

## 4. Discussion

In our population, the prevalence of MBCs and SBCs resulted, respectively, 7% and 3%. We did not find significant differences in terms of OS among MBCs, SBCs, and UBCs. In particular no cancer-related mortality was observed among MBC and SBC patients, and there was no significant difference in death for other causes among the three groups. In our meta-analysis, a significantly more favourable outcome, in terms of longer survival, was observed in UBCs than in MBCs or SBCs. Rank correlation test of funnel plot asymmetry was not significant in all the performed analysis excluding possible publication bias.

### 4.1. MBC and SBC Prevalence

The incidence of MBC and SBC is relatively low, ranging in the literature from about 1% to as high as 21% [4, 27–30]. In our population, the SBCs prevalence resulted comparable to that described by other authors (Figure 2), but the meta-analysis of MBCs prevalence shows a great variability among the different studies (Figure 2), probably due to the significant difference of follow-up length and to the general disagreement about the definition of “metachronous” cancer (Table 6). In fact, the different authors defined as “metachronous” those tumors appearing after different time intervals from the first BC diagnosis, starting from a month and up to 5 years [31, 32]. In the more recent published studies, 3, 6, and 12 months are the most frequently considered time cutoffs to divide between SBCs and MBCs. The distinction between “metachronous” and “synchronous” is only time dependent and despite an evident synchronicity of the clinical appearance, which probably corresponds to a similarity in the time of cell transformation beginning, there may be a synchronicity of presence at the time of the first diagnosis even in the absence of any clinical or imaging evidence of the second tumor that leads this tumor to be defined clinically as “metachronous.” Therefore, although most of the authors define as “synchronous” breast cancers which present between the time of the primary tumor diagnosis and one year from it [7, 15, 17–19, 25, 26], Bloom and colleagues extended the “synchronous” BC definition to five years from the first diagnosis [32]. In our study, we chose the “synchronous” definition for every cancer presenting within 6 months from the original diagnosis.

In accordance with the literature, MBCs represented the majority of multiple breast cancers in our retrospective study (7% versus 3%) and affected women were younger at the time of the first diagnosis than others [7], whereas SBCs appeared in elderly women [33]. A possible explanation for such age difference may be the longer life expectancy of younger women with tumors of favorable prognosis who are therefore at high risk of developing a second breast malignancy [34]. Another consideration that could justify the older age in SBCs than MBCs or other cancers is that many older women neglect their health and find out their disease (or better accept the disease they already found out) in a more advanced stage, so that it may be just more statistically probable to detect another breast cancer (which in other circumstances would have been considered metachronous).

### 4.2. MBC and SBC Characteristics

MBCs presented higher grading and lower hormone receptors expression than UBCs. Meanwhile, SBCs presented higher hormone receptors expression than UBCs and lower grading than MBCs. The higher prevalence of estrogen receptors positivity in SBCs is confirmed also by the literature [33]. Anyway, both MBCs and SBCs had higher incidence of some histological negative prognostic factors (lobular invasive histology, high grading, multifocality, and lymph node extracapsular invasion).

Our data about the tumor hormonal status and histology demonstrate a lower concordance between the two tumors than the literature. In fact, Kheirelseid and colleagues found out a histological and ER-status concordance between the two BC diagnoses of, respectively, 79.2% and 49.5% [33]. Renz and colleagues demonstrated a histological concordance of 54.8%, ER and PR status concordance of 86.2% and 79.3%, and also a great similarity between the magnetic resonance imaging (MRI) features of the two breast cancers [35]. The concordance in hormone receptor status of first and second breast cancers affecting the same patient could suggest that the two tumors may arise in a common milieu and that their subtypes are predetermined in the early stage of breast cancer development [36, 37].

The current literature suggests also the following predictive factors for bilateral breast cancer development: BC familial history [7, 25, 27], BRCA gene mutations [38], HER-2/Neu positivity [39], overweight [40], lobular histology [3, 41], and metropolitan residence, being this last probably due to a better access to the mammographic screening [25]. In fact, also in our study second MBCs and SBCs were more likely to be discovered by mammography.

### 4.3. Interval between First and Second MBC

In our study we confirmed the previous literature where the prevalence of MBCs after 10 years from the original diagnosis was around the 40% [33]. Such long time risk of MBC, which did not diminish with the pass of time, underlies the crucial role of prolonged ipsi- and contralateral mammographies in the postoperative follow-up of these patients [5, 33]. Moreover, some authors suggest that the annual breast ultrasonography could be a useful adjunctive tool to mammography for the detection of MBCs [42].

Hartman and colleagues demonstrated that the mortality rate of multiple breast cancers is inversely proportional to the age and to the time interval between the first and the second diagnosis in case of MBC [1]. In particular, it is even 120% higher in SBC women younger than 50, whereas it is only 40% higher in the same women after the 50th year of age, and, in case of MBC, it decreases with the passing of follow-up, so that women with a diagnosis of MBC more than 10 years after the first diagnosis result to have a 5-year cancer mortality not significantly different from that of women of the same age with a UBC. In our population, a shorter time interval between the first and the second tumor in women affected by MBC resulted to be associated with a significantly lower hormone receptors positivity (*P* < 0.05), higher grading, multifocality, extended in situ component, and peritumoral vascular invasion (p n.s.). And actually, in terms of biological aggressiveness, very close MBCs resemble SBCs, which the literature demonstrates to have a more aggressive histological type and to be poorly differentiated [37] and at greater risk for distant metastasis than UBCs [1, 15, 37]. Anyway, in our population, a shorter time interval between the two breast cancers did not predict a worse OS in MBC patients, as described also by other authors [33, 43].

### 4.4. MBC and SBC Survival

Most of the authors agree that no significant difference exists in survival for patients with unilateral compared to all bilateral breast cancers [39] or compared to MBC diagnosed after 5 years [7]. On the contrary, SBCs are associated with poorer survival in comparison to both MBCs and UBCs [7, 15, 27, 39], and only few studies describe an inverse tendency of better survival in case of SBC compared to MBC [19, 25].

Although our meta-analysis demonstrates a significant better outcome in UBCs than SBCs or MBCs, our retrospective study revealed no statistically significant difference in the OS among MBCs, SBCs, and UBCs, but we found better DFS in UBCs than MBCs or SBCs (Figure 1). This result could be justified by a more aggressive therapeutic management of women affected by multiple BC, who are more likely to receive a radical mastectomy, and by a more prompt imaging diagnosis of a second breast cancer in women followed up for a first breast lesion. In general, patients affected by MBCs or SCBs are more frequently treated with radical surgery, even if there is no clear demonstration of its usefulness [14, 16].

Finally, taking into consideration the impact of the different therapeutic approaches on SBC and MBC outcome, our data suggest a possible interesting role for the adjuvant hormonal therapies in the prevention or at least in the control of an eventual second primary BC. In fact, in our population, the higher hormone receptors positivity of SBCs and of the second MBCs allowed the women affected by multiple BCs to be more frequently treated with adjuvant hormonal therapies, and the second MBCs usually became evident sometime after stopping the hormonal treatment. Therefore, one could wonder if a radical surgery associated with an intensive, adjuvant chemotherapy or hormonal therapy may significantly improve MBC and SBC outcome. And we think that in order to overcome this question only a multicentric, prospective, clinical trial may try to give an answer.

### 4.5. Pros and Cons

Despite the number of patients we took into consideration is relatively smaller than that of the major, multicentric studies, a strength point of our work is the homogeneity of our population, as well as the reliability of the diagnostic and therapeutic management. Moreover, we chose to take into consideration only breast cancer diagnosed within 2004, in order to have an adequate follow-up length to more accurately evaluate patients outcome. Finally, in our study, we present for the first time a meta-analysis about survival statistics about MBCs and SBCs that are a rare entity which could not be easily investigated in single center studies.

## 5. Conclusions

In conclusion, despite the MBCs and SBCs relatively low prevalence, the presence of a long time risk of MBC confirms the need of ipsi- and contralateral mammographies in the postoperative follow-up of BC patients. Although no significant difference in the OS among MBCs, SBCs, and UBCs was observed, SBCs and second tumors in case of MBCs appear to have a higher prevalence of histological negative prognostic factors, to have a worse DFS, and to receive more frequently radical surgery. In our meta-analysis UBCs had better outcomes than MBCs and SBCs. Furthermore, in our opinion, further studies are required in order to better understand the clinical impact of radical surgery and different medical and hormonal therapies on the outcome of these patients.

## Supplementary Material

Supplemental Figure 1: we show the systematic review and meta-analysis flow diagram. And in Supplemental List 1: we show the included and excluded studies in the meta-analysis.

## Figures and Tables

**Table 1:** Population characteristics: data is presented, where appropriate, as mean value (±standard deviation) and one way ANOVA*, median value (interquartile range) and Kruskall-Wallis test**, or prevalence and chi-square test.

	UBC	1st MBC	2nd MBC	1st SBC	2nd SBC	*P*
Women age (years)*	58.71 (±15.47)	56.54 (±13.68)	64.38 (±13.41)	64.94 (±14.72)	65.25 (±14.15)	<0.05
BMI (kg/m^2^)*	26.74 (±5.24)	26.17 (±6.14)		25.18 (±4.64)		0.556
Time interval between 1st and 2nd MBC (months)**			72 (40–120)			N/A
Follow-up time**	79 (70–85)	97 (82–120)	66 (23–82)	79 (72–90)		<0.05
Tobacco smoke	2.46% (13/528)	0% (0/39)		0% (0/16)		0.500
Familial history	0.95% (5/528)	0% (0/39)		6.25% (1/16)		0.094
Mode of diagnosis						
Unknown	4.55% (24/528)	2.56% (1/39)	2.5% (1/40)	0% (0/16)	0% (0/16)	0.709
Objective examination	46.78% (247/528)	69.23% (27/39)	30% (12/40)	31.25% (5/16)	12.5% (2/16)	<0.05
Mammography	36.17% (191/528)	20.51% (8/39)	57.5% (23/40)	43.75% (7/16)	62.5% (10/16)	<0.05
Ultrasound	12.5% (66/528)	7.69% (3/39)	10% (4/40)	25% (4/16)	25% (4/16)	0.236
Radiotherapy	56.44% (241/427)	64.86% (24/37)		46.67% (7/15)		0.442
Chemotherapy	45.88% (195/425)	47.22% (17/36)		57.14% (8/14)		0.703
Hormonal therapy	70.85% (299/422)	80.56% (29/36)		100% (14/14)		<0.05
Tamoxifen therapy	44.13% (233/528)	53.85% (21/39)		62.5% (10/16)		0.187

**Table 2:** Histology and TNM staging (AJCC/UICC) of the tumors at the time of diagnosis: in this table we present prevalences and *P* values referring to chi-square test.

	UBC	1st MBC	2nd MBC	1st SBC	2nd SBC	*P*
Histology						
Ductal invasive carcinoma	65.91% (348/528)	69.23% (27/39)	65% (26/40)	43.75% (7/16)	68.75% (11/16)	0.447
Lobular invasive carcinoma	8.9% (47/528)	15.38% (6/39)	12.5% (5/40)	37.5% (6/16)	6.25% (1/16)	<0.05
Ductal and lobular invasive carcinoma	13.45% (71/528)	7.69% (3/39)	5% (2/40)	12.5% (2/16)	6.25% (1/16)	0.418
Other invasive carcinomas	3.41% (18/528)	2.56% (1/39)	0% (0/40)	0% (0/16)	6.25% (1/16)	0.645
Ductal in situ carcinoma	7.39% (39/528)	5.13% (2/39)	17.5% (7/40)	6.25% (1/16)	6.25% (1/16)	0.212
Lobular in situ carcinoma	0.38% (2/528)	0% (0/39)	0% (0/40)	0% (0/16)	0% (0/16)	0.981
Unknown	0.57% (3/528)	0% (0/39)	0% (0/40)	0% (0/16)	6.25% (1/16)	0.067
Tumor (UICC)						
Tis-T1	67.94% (356/524)	68.42% (26/38)	70% (28/40)	62.5% (10/16)	75% (12/16)	0.957
T2	24.43% (128/524)	26.32% (10/38)	22.5% (9/40)	37.5% (6/16)	18.75% (3/16)	0.755
T3-T4	7.63% (40/524)	5.26% (2/38)	7.5% (3/40)	0% (0/16)	6.25% (1/16)	0.806
Lymph nodes (UICC)						
N0	66.86% (347/519)	75.68% (28/37)	70% (28/40)	56.25% (9/16)	93.75% (15/16)	0.122
N1	19.27% (100/519)	16.22% (6/37)	15% (6/40)	18.75% (3/16)	6.25% (1/16)	0.690
N2	7.51% (39/519)	2.7% (1/37)	10% (4/40)	12.5% (2/16)	0% (0/16)	0.476
N3	6.36% (33/519)	5.41% (2/37)	5% (2/40)	12.5% (2/16)	0% (0/16)	0.679

**Table 3:** Other characteristics of the tumors: in this table we present prevalences and *P* values referring to chi-square test.

	UBC	1st MBC	2nd MBC	1st SBC	2nd SBC	*P*
Grading						
G1	7.16% (35/489)	7.69% (3/39)	7.5% (3/40)	12.5% (2/16)	18.75% (3/16)	0.480
G2	67.48% (330/489)	41.03% (16/39)	55% (22/40)	81.25% (13/16)	68.75% (11/16)	<0.05
G3	25.36% (124/489)	51.28% (20/39)	37.5% (15/40)	6.25% (1/16)	12.5% (2/16)	<0.05
ER positivity	73.3% (387/528)	51.28% (20/39)	67.5% (27/40)	75% (12/16)	81.25% (13/16)	<0.05
PR positivity	68.94% (364/528)	43.59% (17/39)	60% (24/40)	75% (12/16)	75% (12/16)	<0.05
HER-2/Neu positivity	8.9% (47/528)	7.69% (3/39)	12.5% (5/40)	6.25% (1/16)	6.25% (1/16)	0.912
Mib-1/Ki-67 (%)						
1 to 20	34.98% (85/243)	57.14% (4/7)	32% (8/25)	57.14% (4/7)	60% (3/5)	0.376
20 to 30	18.11% (44/243)	0% (0/7)	12% (3/25)	0% (0/7)	0% (0/5)	0.331
>30	46.91% (114/243)	42.86% (3/7)	56% (14/25)	42.86% (3/7)	40% (2/5)	0.911
Multifocality	26.7% (141/528)	5.13% (2/39)	27.5% (11/40)	50% (8/16)	18.75% (3/16)	<0.05
Extended in situ component	22.16% (117/528)	17.95% (7/39)	32.5% (13/40)	25% (4/16)	0% (0/16)	0.110
Peritumoral vascular invasion	6.82% (36/528)	5.13% (2/39)	7.5% (3/40)	6.25% (1/16)	0% (0/16)	0.850
Comedo-like necrosis	8.9% (47/528)	2.56% (1/39)	12.5% (5/40)	6.25% (1/16)	6.25% (1/16)	0.581
Lymph node isolated tumor cells	3.6% (19/528)	0% (0/39)	0% (0/40)	0% (0/16)	0% (0/16)	0.390
Lymph node extracapsular invasion	2.27% (12/528)	0% (0/39)	0% (0/40)	12.5% (2/16)	0% (0/16)	<0.05

**Table 4:** Crude and adjusted* hazard ratios (HR) and 95% confidence intervals (CI) for SBC and MBC and the risk of locoregional recurrence and distant metastasis in comparison with UBC (Cox proportional hazards model).

	HR (95% CI)	*P*	HR (95% CI)*	*P*
UBC	1		1	
MBC	2.03 (1.01–4.08)	<0.05	2.70 (1.04–7.03)	<0.05
SBC	0.84 (0.21–3.43)	0.809	1.42 (0.33–6.21)	0.639

**Table 5:** Characteristics of second MBC stratified per time interval after diagnosis of first MBC. We considered as cutoff the median time interval that was 47 months. In this table we present mean values (±standard deviation) and one way ANOVA* or prevalences and chi-square test.

	≤47 moths	>47 months	*P*
Women age (years)*	63.14 (±14.22)	54.67 (±13.47)	<0.05
BMI (kg/m^2^)*	25.2 (±4.01)	26.6 (±7.08)	0.448
Multifocality	28.57% (6/21)	26.32% (5/19)	0.873
Extended in situ component	42.86% (9/21)	21.05% (4/19)	0.141
Peritumoral vascular invasion	9.52% (2/21)	5.26% (1/19)	0.609
ER positivity	57.14% (12/21)	78.95% (15/19)	0.141
PR positivity	42.86% (9/21)	78.95% (15/19)	<0.05
HER-2/Neu positivity	14.29% (3/21)	10.53% (2/19)	0.720
Histology			
Ductal invasive carcinoma	61.9% (13/21)	68.42% (13/19)	0.666
Lobular invasive carcinoma	23.81% (5/21)	0% (0/19)	<0.05
Ductal and lobular invasive carcinoma	4.76% (1/21)	5.26% (1/19)	0.942
Ductal in situ carcinoma	9.52% (2/21)	26.32% (5/19)	0.163
Grading			
G1	14.29% (3/21)	0% (0/19)	0.087
G2	47.62% (10/21)	63.16% (12/19)	0.324
G3	38.1% (8/21)	36.84% (7/19)	0.935
Mib-1/Ki-67 (%)			
1 to 20	43.75% (7/16)	11.11% (1/9)	0.093
20 to 30	12.5% (2/16)	11.11% (1/9)	0.918
>30	43.75% (7/16)	77.78% (7/9)	0.100

**Table 6:** Description of the studies included in our meta-analysis.*Mean, **median, and ^¶^follow-up divided between SBC, MBC, and UBC.

Study	MBC/SBC	MBC after (months)	Follow-up years	Study design	Years considered	Follow-up until	Months between metachronous
Heron et al. 2000 [15]	MBC/SBC	12	5/7/3^∗∗¶^	Hosp.-based	60/95	′95	44
Polednak 2003 [26]	SBC	3	—	Pop.-based	95/99	′00	NA
Takahashi et al. 2005 [19]	MBC/SBC	6	7*	Hosp.-based	60/01	′01	113
Verkooijen et al. 2007 [18]	MBC/SBC	6	7**	Pop.-based	70/02	′02	80
Kuo et al. 2009 [17]	MBC/SBC	6	8*	Hosp.-based	90/99	′04	36
Vuoto et al. 2010 [7]	MBC/SBC	12	8/13/—^∗¶^	Hosp.-based	70/07	′07	81
Beckmann et al. 2011 [25]	MBC/SBC	3	6*	Pop.-based	97/07	′09	34

## Abbreviations

BC: Breast cancer
MBC: Metachronous breast cancer
SBC: Synchronous breast cancer
UBC: Unilateral breast cancer
OS: Overall survival
TNM: Tumor, node, and metastasis
AJCC/UICC: American Joint Commission on Cancer/Union Internationale Contre le Cancer.
