# Supplementary material for: Synchronous and Metachronous Breast Malignancies: A Cross-Sectional Retrospective Study and Review of the Literature
Source: Biomed Res Int. 2014 Apr 27;2014:250727. doi: 10.1155/2014/250727 (PMC4022260; doi:10.1155/2014/250727)
Supplement: Supplementary file 1 — Supplemental Figure 1: we show the systematic review and meta-analysis flow diagram. And in Supplemental List 1: we show the included and excluded studies in the meta-analysis. [file 250727.f1.pdf]

Supplemental List 1

INCLUDED STUDIES: fulfilled the requirements.

1)Heron, D. E.; Komarnicky, L. T.; Hyslop, T.; Schwartz, G. F. & Mansfield, C. M. (2000), 'Bilateral breast carcinoma: risk factors and outcomes for patients with synchronous and metachronous disease.', *Cancer* **88**(12), 2739—2750.

2)Polednak, A. P. (2003), 'Bilateral synchronous breast cancer: a population-based study of characteristics, method of detection, and survival.', *Surgery* **133**(4), 383--389.

3)Takahashi, H.; Watanabe, K.; Takahashi, M.; Taguchi, K.; Sasaki, F. & Todo, S. (2005), 'The impact of bilateral breast cancer on the prognosis of breast cancer: a comparative study with unilateral breast cancer.', *Breast Cancer* **12**(3), 196--202.

4)Verkooijen, H. M.; Chatelain, V.; Fioretta, G.; Vlastos, G.; Rapiti, E.; Sappino, A.-P.; Bouchardy, C. & Chappuis, P. O. (2007), 'Survival after bilateral breast cancer: results from a population-based study.', *Breast Cancer Res Treat* **105**(3), 347--357.

5)Kuo, W.-H.; Yen, A. M.-F.; Lee, P.-H.; Chen, K.-M.; Wang, J.; Chang, K.-J.; Chen, T. H.-H. & Tsau, H.-S. (2009), 'Cumulative survival in early-onset unilateral and bilateral breast cancer: an analysis of 1907 Taiwanese women.', *Br J Cancer* **100**(4), 563--570.

6)Vuoto, H. D.; García, A. M.; Candás, G. B.; Zimmermann, A. G.; Uriburu, J. L.; Isetta, J. A. M.; Cogorno, L.; Khoury, M. & Bernabó, O. L. (2010), 'Bilateral breast carcinoma: clinical characteristics and its impact on survival.', *Breast J* **16**(6), 625--632.

7)Beckmann, K. R.; Buckingham, J.; Craft, P.; Dahlstrom, J. E.; Zhang, Y.; Roder, D. & Stuart-Harris, R. (2011), 'Clinical characteristics and outcomes of bilateral breast cancer in an Australian cohort.', *Breast* **20**(2), 158--164.

EXCLUDED STUDIES: because not focused on the problem, impossible to retrieve complete article (and the abstract information not enough detailed), or not possible to retrieve enough information for HR considering outcome death of MBC vs UBC or of SBC vs UBC.

1)Kollias, J.; Ellis, I. O.; Elston, C. W. & Blamey, R. W. (2001), 'Prognostic significance of synchronous and metachronous bilateral breast cancer.', *World J Surg* **25**(9), 1117--1124.

2)Carmichael, A. R.; Bendall, S.; Lockerbie, L.; Prescott, R. & Bates, T. (2002), 'The long-term outcome of synchronous bilateral breast cancer is worse than metachronous or unilateral tumours.', *Eur J Surg Oncol* **28**(4), 388--391.

3)Claus, E. B.; Stowe, M.; Carter, D. & Holford, T. (2003), 'The risk of a contralateral breast cancer among women diagnosed with ductal and lobular breast carcinoma in situ: data from the Connecticut Tumor Registry.', *Breast* **12**(6), 451--456.

4)Jobsen, J. J.; van der Palen, J.; Ong, F. & Meerwaldt, J. H. (2003), 'Synchronous, bilateral breast cancer: prognostic value and incidence.', *Breast* **12**(2), 83--88.

5)Levi, F.; Randimbison, L.; Te, V.-C. & Vecchia, C. L. (2003), 'Prognosis of bilateral synchronous breast cancer in Vaud, Switzerland.', *Breast* **12**(2), 89--91.

6)Gao, X.; Fisher, S. G. & Emami, B. (2003), 'Risk of second primary cancer in the contralateral breast in women treated for early-stage breast cancer: a population-based study.', *Int J Radiat Oncol Biol Phys* **56**(4), 1038--1045.

7)Intra, M.; Rotmensz, N.; Viale, G.; Mariani, L.; Bonanni, B.; Mastropasqua, M. G.; Galimberti, V.; Gennari, R.; Veronesi, P.; Colleoni, M.; Tousimis, E.; Galli, A.; Goldhirsch, A. & Veronesi, U. (2004), 'Clinicopathologic characteristics of 143 patients with synchronous bilateral invasive breast carcinomas treated in a single institution.', *Cancer* **101**(5), 905--912.

8)Mertens, W. C.; Hilbert, V. & Makari-Judson, G. (2004), 'Contralateral breast cancer: factors associated with stage and size at presentation.', *Breast J* **10**(4), 304--312.

9)Khairy, G. A.; Guraya, S. Y.; Ahmed, M. E. & Ahmed, M. A. (2005), 'Bilateral breast cancer. Incidence, diagnosis and histological patterns.', *Saudi Med J* **26**(4), 612--615.

10)Hartman, M.; Czene, K.; Reilly, M.; Adolfsson, J.; Bergh, J.; Adami, H.-O.; Dickman, P. W. & Hall, P. (2007), 'Incidence and prognosis of synchronous and metachronous bilateral breast cancer.', *J Clin Oncol* **25**(27), 4210--4216.

11)Kilciksiz, S.; Gokce, T.; Baloglu, A.; Calli, A.; Kaynak, C.; Kilic, B.; Eski, E.; Nalbantoglu, G. & Yigitbas, H. A. (2007), 'Characteristics of synchronous- and metachronous-type multiple primary neoplasms: a study of hospital-based cancer registry in Turkey.', *Clin Genitourin Cancer* **5**(7), 438--445.

12)Quan, G.; Pommier, S. J. & Pommier, R. F. (2008), 'Incidence and outcomes of contralateral breast cancers.', *Am J Surg* **195**(5), 645--50; discussion 650.

13)Yadav, B. S.; Sharma, S. C.; Patel, F. D.; Ghoshal, S. & Kapoor, R. K. (2008), 'Second primary in the contralateral breast after treatment of breast cancer.', *Radiother Oncol* **86**(2), 171--176.

14)Díaz, R.; Munárriz, B.; Santaballa, A.; Palomar, L. & Montalar, J. (2010), 'Synchronous and metachronous bilateral breast cancer: a long-term single-institution experience.', *Med Oncol*.

15)Schmid, S. M.; Pfefferkorn, C.; Myrick, M. E.; Viehl, C. T.; Obermann, E.; Schötzau, A. & Güth, U. (2011), 'Prognosis of early-stage synchronous bilateral invasive breast cancer.', *Eur J Surg Oncol* **37**(7), 623--628.

16)Kheirelseid, E. A. H.; Jumustafa, H.; Miller, N.; Curran, C.; Sweeney, K.; Malone, C.; McLaughlin, R.; Newell, J. & Kerin, M. J. (2011), 'Bilateral breast cancer: analysis of incidence, outcome, survival and disease characteristics.', *Breast Cancer Res Treat* **126**(1), 131--140.

17)Vichapat, V.; Gillett, C.; Fentiman, I. S.; Tutt, A.; Holmberg, L. & Lüchtenborg, M. (2011), 'Risk factors for metachronous contralateral breast cancer suggest two aetiological pathways.', *Eur J Cancer*.

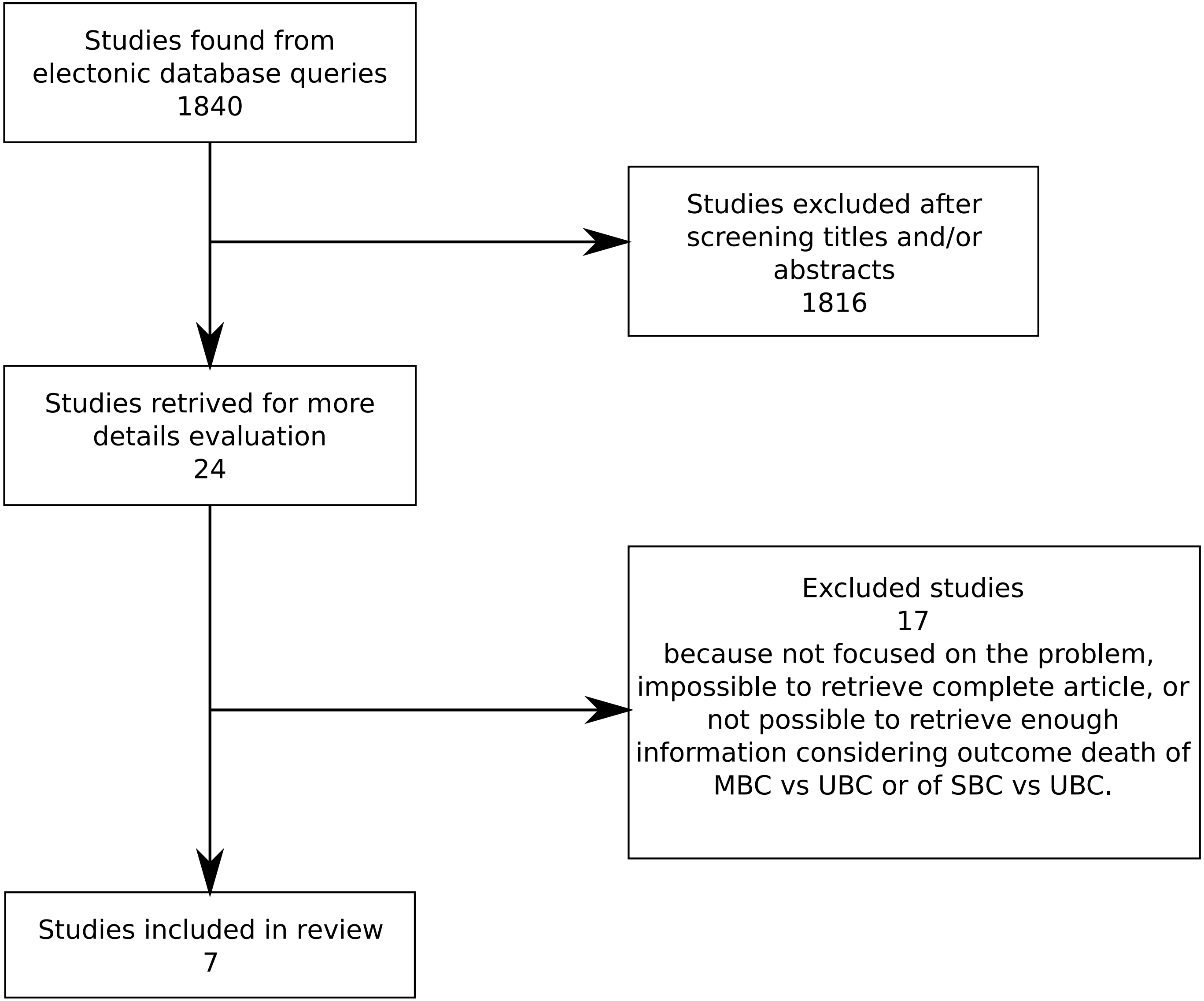
Studies found from electonic database queries 1840
Studies excluded after screening titles and/or abstracts 1816
Studies retrived for more details evaluation 24
Excluded studies 17 because not focused on the problem, impossible to retrieve complete article, or not possible to retrieve enough information considering outcome death of MBC vs UBC or of SBC vs UBC.
Studies included in review 7
